# Effect of oval foramen restriction and premature contraction of the arterial catheter on right heart function of fetuses and infants

**DOI:** 10.7717/peerj.14702

**Published:** 2023-01-04

**Authors:** Hongqiang Liu, Jinqiu Li, Xiaolong Cao, Yicheng Wang, Dehui Wen, Fengqun Dong, Jing Wang, Tian Li

**Affiliations:** 1 The First Affiliated Hospital of Hebei North University, Zhangjiakou, China; 2 Department of Aerospace Medicine, Fourth Military Medical University, Xi’an, China; 3 Hebei Maternity Hospital, Shijiazhuang, China; 4 Life Science Research Center, Hebei North University, Zhangjiakou, China; 5 School of Basci Med, Fouth Military Medical University, Xi’an, China

**Keywords:** Oval foramen restriction, Premature contraction, Arterial catheter, Right heart function, Fetuses, Infant

## Abstract

**Objective:**

The effect of fetal oval foramen restriction and premature contraction of the arterial catheter for the right heart function of fetuses and infants was studied by evaluating the right and left ventricular (RV/LV) ratios, the tricuspid annular plane systolic excursion (TAPSE) value, and the Tei index of right heart function parameters.

**Methods:**

This study was approved by the Ethics Committee of First Affiliated Hospital of Hebei North University (K20190116). We collected 257 fetuses between March 2020 and December 2021. Among these, 98 fetuses that did not have any heart abnormalities were assigned to group A, 91 fetuses with restriction of the left and right atrial channels were assigned to group B, and 68 fetuses with premature contraction of the arterial catheter were assigned to group C. The ventricular transverse diameter, the right heart TAPSE value and the Tei index of fetuses in late pregnancy and 90 days after birth were measured in the three groups, and the diagnostic value of each index for the right heart function injury was evaluated. *P* < 0.05 indicates significant.

**Results:**

The P-value of the TAPSE value and Tei index of infants in BC and AC groups and postnatal infants were less than 0.05, which was significant. In the BC group, the RV/LV ratio of fetuses was compared when *P* > 0.05, which was not significant; however, *P* < 0.05 after birth was considered significant. For fetuses and postnatal infants in the BC group, the RV/LV ratio was negatively associated with the TAPSE value. However, it was positively associated with the Tei index; Diagnostic test results. To predict impaired right heart function after birth, TAPSE had low diagnostic value, RV/LV and Tei index had high diagnostic value.

**Conclusions:**

Oval foramen restriction and premature contraction of the arterial catheter may affect the right heart function after birth and be related to the degree of the right heart enlargement. Although TAPSE prediction of the fetal and postnatal right heart function is limited, the RV/LV ratio and the Tei index can be used to predict impaired right heart function after birth.

## Introduction

Theoval foramen and valves form important blood flow channels in the left and right atria during the fetal period. This provides about 76% of the left heart blood flow, plays an important role in promoting the left heart development, and maintains the left and right heart pressure. However, a restriction in this channel will increase the right heart volume load (Furtado et al., 2012; Tulzer et al., 2020). The arterial catheter is open during the fetal period and is an important channel between the pulmonary artery and the descending aorta. It constitutes more than 80% of the blood flow source of the descending aorta. Therefore, premature contraction of the arterial catheter significantly aggravates the pressure load of the right heart (Alvarez & McBrien, 2018; Enzensberger et al., 2012).

The two factors above are the main causes of right heart enlargement in the fetal period. Furthermore, whether the right heart enlargement should be clinically managed the same way as other conditions and the extent to which the right heart enlargement may affect the right heart function after birth is a dilemma that we encounter in clinical settings. Hence, we aimed to obtain effective parameters that can predict impaired right heart function after birth by measuring the right and left ventricular (RV/LV) ratios, tricuspid annular plane systolic excursion tricuspid annular plane systolic excursion (TAPSE) value, and the Tei index. These provide reliable imaging information to make favorable clinical choices for pregnant women and their fetuses.

## Materials and methods

### Data collection

This study was approved by the Ethics Committee of First Affiliated Hospital of Hebei North University (K20190116), and all pregnant women gave written informed consent for participating this study. Because the collection of patient information is incomplete, patients quitted midway and the collected images cannot be analyzed and other reasons, finally, two hundred and fifty-seven complete cases were collected between March 2020 and December 2021; 98 fetuses that did not have any heart abnormalities were included in group A, pregnant women were aged 22–35 years, the mean age was 28.93 ± 4.75 years, the fetal gestational age occurred between 28 and 39 weeks, and the average gestational age was 32.45 ± 2.47 weeks. Ninety-one fetuses with restrictions were included in group B. Pregnant women were aged 21–35 years, the mean age was 30.14 ± 4.05 years, the fetal gestational age occurred between 28 and 39 weeks, and the mean gestational age was 33.02 ± 2.34 weeks. Sixty-eight fetuses with premature arterial catheter constriction were included in group C. Pregnant women were aged 21–35 years, the mean age was 30.13 ± 4.28 years, the fetal gestational age occurred between 28 and 39 weeks, and the average gestational age was 32.97 ± 2.70 weeks. The TAPSE value and Tei index of the ventricular transverse diameter and right heart of the three groups were measured during late pregnancy and at about 90 days after birth. The inclusion criteria were as follows: (1) late pregnancy singleton; (2) an accurate gestational age; (3) pregnant women who have no history of heart, kidney, or metabolic diseases. The exclusion criteria were as follows: (1) Fetal abnormalities were found during and after fetus; (2) persistent arrhythmia and myocardial disease; (3) inability to obtain ideal ultrasound images; (4) unavailability of complete information.

### Instruments and methods

**Figure 1:**
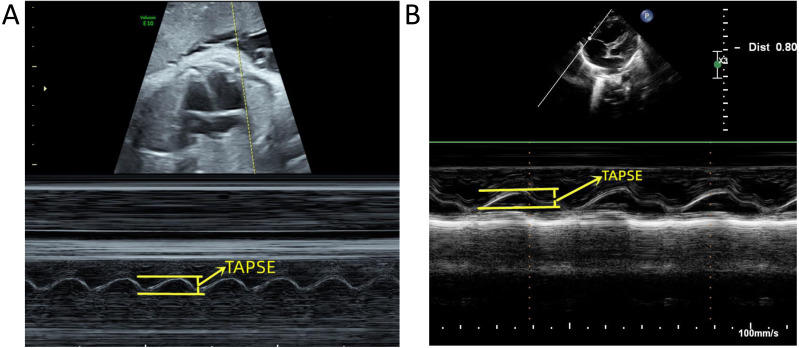
Measurement of tricuspid annular plane systolic excursion (TAPSE). (A) Measurement of TAPSE before birth. (B) Measurement of TAPSE after birth.

**Figure 2:**
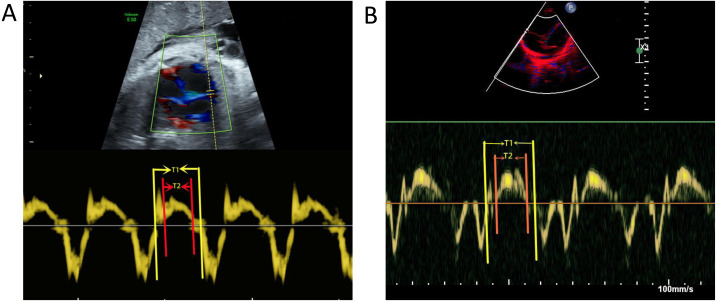
Measurement of Tei index. (A) Measurement of Tei index before birth. (B) Measurement of Tei index after birth. T1: Tricuspid valve opening to closure time; T2: Right ventricular ejection time.

A PhilipsEPIQ7C diasonograph (Philips, Amsterdam, the Netherlands) was used with a C5-1 probe of frequency 1 to 5 MHz and an S5-1 probe of frequency 1 to 5 MHz. A GE VolusonE10 diasonograph (GE, Tiefenbach, Austria), C5-1 probe with frequency 1 to 5 MHz, and C9-2 probe with frequency 2 to 9 MHz were used for the following: (1) routine fetal ultrasound examination excluding malformations and verified fetal gestational age; (2) fetal echocardiography excluding structural abnormalities in the fetal heart; (3) measurement of the TAPSE value, showing the standard four-chambered heart. The position of the probe was adjusted to move the tip forward or backward, the M-type ultrasound mode was initiated, the sampling line was placed at the tangent position at the junction of the anterior lobe ring and the right ventricular free wall, the angle of the longitudinal movement was adjusted to < 15°, and the distance from the lowest point of movement to the highest was measured (Figs. 1A, 1B); (4) tissue doppler imaging (TDI) was obtained by the tricuspid ring movement spectrum of the mitral lobe at the anterior lobe and the valve ring junction. The scan speed was adjusted to the maximum, and the Tei index was measured (Figs. 2A, 2B). All data were measured for the three stable cardiac cycles, and the averages were recorded.

### Statistical methods

SPSS 26.0 software (version 26.0, IBM, Armonk, NY, USA) was used for statistical data analysis; measurement data were indicated by (x ±). Correlation between parameters was analyzed using Pearson analysis. ROC (receiver operating characteristic) curves were used to test the accuracy of the diagnostic tests, and an independent sample *t*-test was used for the data comparison ratio of each group. The values were set as *α* = 0.05, *P* < 0.05; differences were considered statistically significant.

## Results

### Comparison of basic data

The age of the pregnant women, gestational age at fetal echocardiography, and echocardiography examination days of infants after birth in groups A, B, and Care presented in Table 1. The difference in the basic data of the three groups was not significant (*P* > 0.05, Table 2).

**Table 1:** General data of the cases in each group.

Group	Example number (n)	Pregnant woman age (years)	Fetal gestational weeks (week)	Birth examination days (day)
A	98	28.93 ± 4.75	32.45 ± 2,47	92.01 ± 1.56
B	91	30.14 ± 4.05	32.86 ± 2.02	92.26 ± 1.76
C	68	30.13 ± 4.28	32.97 ± 2.70	92.25 ± 1.62

**Table 2:** Comparison of the general data.

Comparison item	group	F	P
Pregnant woman age	AB	6.567	0.063
	AC	3.494	0.099
	BC	0.158	0.987
Fetal pregnancy	AB	5.229	0.217
	AC	0.183	0.200
	BC	5.617	0.762
Birth examination days	AB	2.047	0.294
	AC	0.761	0.338
	BC	0.261	0.960

### Comparison of the right heart function parameters before and after birth

The TAPSE value before and after birth was higher in all the groups: A, B, and C. In contrast, the Tei index was lower in groups A, B, and C. The difference in the parameters of TAPSE and Tei index was significant at before and after birth (*P* < 0.05). The RV/LV ratio before birth was higher in group B than in C (*P* > 0.05), and the difference was not significant. However, the RV/LV ratio after birth was lower in group B than in C (*P* < 0.05), and the difference was considered significant.

### Association analysis

Regarding fetal and postnatal measurements in group B, the larger the TAPSE value before birth, the lower were the Tei index value. Furthermore, the overall distribution density was roughly uniform, and the change direction was the same. The larger the postnatal TAPSE value, the lower the Tei index value. Regions with a higher overall distribution density were located in the regions with larger TAPSE values, and the change direction was the same. The magnitude of Pearson correlation coefficients with the TAPSE value and Tei index were −0.794 and −0.822, respectively, before and after birth. The two-sided test of the correlation coefficient was significant (*P* < 0.01); however, a negative correlation was observed. The larger the TAPSE value before birth, the lower the RV/LV ratio. A rough uniform overall distribution density and the same change direction were observed. In contrast, the lower the RV/LV ratio, the more were the regions with higher overall distribution density change in the same direction. The magnitude of Pearson correlation coefficients between the TAPSE value and RV/LV ratio were−0.875 and −0.786, respectively, before and after birth. The two-sided test of the correlation coefficient was significant (*P* < 0.01), and a negative correlation was observed—conversely, the larger were the Tei index values before birth, the larger the RV/LV values. The overall distribution density was roughly uniform, and the change direction was the same. The greater the Tei index value after birth, the larger was the RV/LV ratio. Regions with a higher overall distribution density were located in the regions with smaller Tei index values, and the change direction was the same. The magnitude of Pearson correlation coefficients between the Tei index and RV/LV ratio were 0.893 and 0.805, respectively, before and after birth. The two-sided test of the correlation coefficient was significant (*P* < 0.01), and a positive correlation was observed.

The measurements for fetuses before and after birth in group C, the larger the TAPSE value before birth, the lower the Tei index value. The overall distribution density was roughly uniform, and the change direction was the same. The larger the TAPSE value after birth, the lower was the Tei index value; regions with a higher overall distribution density were located in the regions with larger TAPSE values, and the change direction was the same. The magnitude of Pearson correlation coefficients before and after birth between the TAPSE value and Tei index were 0.866 and 0.945, respectively, before and after birth. The two-sided test of the correlation coefficient was significant (*P* < 0.01), and a negative correlation was observed. The larger the TAPSE values before birth, the lower was the RV/LV ratio; the overall distribution density was roughly uniform, and the change direction was the same. The larger the TAPSE value after birth, the lower was the RV/LV ratio; regions with a higher overall distribution density were located in the regions with larger TAPSE values, and the change direction was the same. The magnitude of Pearson correlation coefficients between the TAPSE value and RV/LV ratio were 0.869 and 0.952, respectively, before and after birth. The two-sided test of the correlation coefficient was significant (*P* < 0.01), and a negative correlation was observed. The larger the Tei index values before birth, the larger were the RV/LV ratios; the overall distribution density was roughly uniform, and the change direction was the same. The larger the Tei index value after birth, the larger were the RV/LV ratios; regions with a higher overall distribution density were located in the regions with smaller Tei index values, and the change direction was the same. The magnitude of the Pearson correlation coefficient between the Tei index and RV/LV ratios were 0.873 and 0.919, respectively, before and after birth. The two-sided test of the correlation coefficient was significant (*P* < 0.01) and both were positively correlated.

### Accuracy inspection of the diagnostic test parameters

**Figure 3:**
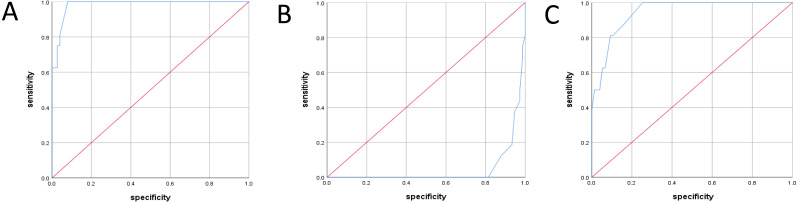
ROC curve of different parameters during fetal period in group B. (A) ROC curves for right and left ventricular (RV/LV) ratio during fetal period. (B) ROC curves for TAPSE during fetal period. (C) ROC curves for Tei index during fetal period.

The diagnostic standard was the fetal right cardiac enlargement after birth. Diagnostic test accuracy inspection in group B: the ROC curve of the RV/LV ratio during the fetal period rose vertically from the lower-left corner to the top line extending from the horizontal direction to the upper right corner (Fig. 3A). The area under the ROC curve was 0.983 (Table 3); if the cut-off value was 1.69, the sensitivity was 84.5%, and the specificity was 94.7%, with a high diagnostic value. The ROC curve of the TAPSE value during the fetal period was located on the right-side and zoomed up (Fig. 3B), and the area under the ROC curve was 0.045 (Table 3), with a low diagnostic value. The Tei index curve in the fetal period rose vertically from the lower-left corner to the top line and extended from the horizontal direction to the upper right corner (Fig. 3C). The area under the ROC curve was 0.944 (Table 3); if the cut-off value was 0.49, the sensitivity was 81.3%, and the specificity was 90%, with a high diagnostic value.

**Figure 4:**
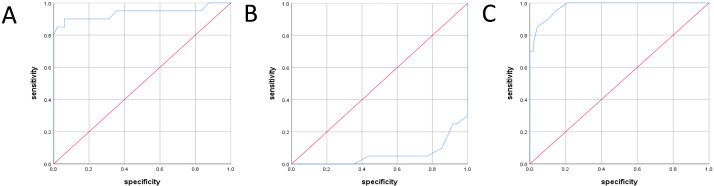
ROC curve of different parameters during fetal period in group C. (A) ROC curves for RV/LV ratio during fetal period. (B) ROC curves for TAPSE during fetal period. (C) ROC curves for Tei index during fetal period.

Diagnostic test accuracy inspection in group C: the ROC curve of the RV/LV ratio during the fetal period rose vertically from the lower-left corner to the top line and extended from the horizontal direction to the top right (Fig. 4A). The area under the ROC curve was 0.937 (Table 4); if the cut-off value was 1.66, the sensitivity was 85.0%, and the specificity was 97.8%, with a high diagnostic value. The ROC curve of the TAPSE value during the fetal period was located on the right side and extended upwards (Fig. 4B). The area under the ROC curve was 0.058 (Table 4), with a low diagnostic value. The Tei index curve in the fetal period rose from the lower-left corner to the top line and extended from the horizontal direction to the top right corner (Fig. 4C). The area under the ROC curve was 0.977 (Table 4); if the cut-off was0.47, the sensitivity was 95.0%, and the specificity was 85.2%, with a high diagnostic value.

**Table 3:** Area under the curve of each inspection variable in group B.

Test result variable		Area	Standard error^a^	Progressive Sig.^b^	Progressive 95% confidence interval	
					lower limit	high lines
Dimension 0	RV/LV	0.983	0.011	0.000	0.962	1.000
	TAPSE	0.045	0.020	0.000	0.006	0.085
	Tei index	0.944	0.024	0.000	0.898	0.990

**Notes.**

a Under the nonparametric assumption.

b Null hypothesis: actual area = 0.5.

**Table 4:** Area under the curve of the each inspection variable in group C.

Test result variable		area	Standard error^a^	Progressive Sig.^b^	Progressive 95% confidence interval	
					lower limit	high lines
Dimension 0	RV/LV	0.937	0.044	0.000	0.850	1.000
	TAPSE	0.058	0.033	0.000	0.000	0.123
	Tei index	0.977	0.014	0.000	0.949	1.000

**Notes.**

a Under the nonparametric assumption.

b Null hypothesis: actual area = 0.5.

## Discussion

### Main finding

Cardiovascular diseases remain the leading cause of death worldwide (Gao et al., 2022; Li et al., 2017; Li et al., 2022; Li et al., 2021; Li et al., 2020). The *P*-value of the TAPSE value and Tei index of infants in BC and AC groups and postnatal infants were less than 0.05, which was significant. In the BC group, the RV/LV ratio of fetuses was compared when *P* > 0.05, which was not significant; however, *P* < 0.05 after birth was considered significant. For fetuses and postnatal infants in the BC group, the RV/LV ratio was negatively associated with the TAPSE value. However, it was positively associated with the Tei index; Diagnostic test results: To predict impaired right heart function after birth, TAPSE had low diagnostic value, RV/LV and Tei index had high diagnostic value. Oval foramen restriction and premature contraction of the arterial catheter may affect the right heart function after birth and be related to the degree of the right heart enlargement. Although TAPSE prediction of the fetal and postnatal right heart function is limited, the RV/LV ratio and the Tei index can be used to predict impaired right heart function after birth.

### Interpretation

Data of 361 pregnant women who volunteered for the trial were collected for this study, including178 fetuses that did not have any heart abnormalities. Echocardiography was not performed in 13 cases during late pregnancy, ideal ultrasound images could not be obtained in nine cases, echocardiography was not performed in 58 cases around 90 days after birth, and 98 cases were eventually included in the analysis (98/178). Of the 107 fetuses with oval orifice restriction, DCRV (Double Chamber Right Ventricle) was observed in a case after birth. Echocardiography was not performed in one case during late pregnancy, ideal ultrasound images could not be obtained in three cases, and echocardiography was not performed in 13 cases around 90 days after birth; hence, 91 cases were included in the analysis (91/107). Of 76 fetuses with premature contraction of the arterial catheter, one death was recorded, and ideal ultrasound images could not be obtained in four cases. Echocardiography was not performed in three cases around 90 days after birth; hence, 68 cases were finally included in the analysis (68/76).

The right heart has an advantage during the fetal period because of the size of the right heart is slightly greater and the blood flow of the right heart is greater than that of the left heart. There are two important physiological processes that coordinate blood flow from the right to the left shunt channels to ensure the normal development of the fetus in this period; (1) a channel formed by the oval foramen and the oval valve located between the left and right atria; (2) another channel formed by the arterial catheter, located between the pulmonary artery and descending aorta (Enzensberger et al., 2012; Furtado et al., 2012; Lei et al., 2018; Tague et al., 2017). The restriction between the left and right atria mainly increases the volume load of the right heart while the restriction of the arterial catheter mainly affects the pressure load of the right heart, and both may affect the right heart function of the fetus (Tulzer et al., 2020; Zhang, Haneishi & Liu, 2021). The pulmonary circulation begins to improve gradually after birth, and the pressure on the left and right heart transits to maturity gradually, although this transition takes about 3 months. During this period, the pulmonary artery pressure is higher in the middle and early neonates, and this condition is also known as physiological pulmonary hypertension (Erickson et al., 2019; Patey et al., 2017). During this period, the pressure of the pulmonary artery changes immensely, and the right heart function index is unstable. However, the pressure of the pulmonary artery gets stabilized in approximately 90 days, which is conducive for the accurate measurement of the right heart function index.

To date, there are no defined guidelines for the fetal right heart function measurement. Therefore, we referred to the adult right heart function evaluation standard to deduce the right heart function evaluation for the fetuses. After considering the four aspects of the right heart function measurement comprehensively, *i.e.,* repeatability, the size of the measurement error, the measurement stability, and the reliability of the right heart function evaluation, we finally chose two indicators, namely the TAPSE value and the Tei index. It is believed that the TAPSE value can be used as an important parameter to evaluate the fetal right heart contraction function, and it has good reproducibility and lower requirements for equipment (Herling et al., 2021; Messing et al., 2013). In this study, the numerical measurement was observed to be more dependent on the angle of the sampling line and the flow direction. The longitudinal displacement of the sampling line and the tricuspid valve ring at the sampling point was less than 15°, which ensured the reliability of the measurement data. The Tei index can reflect the overall right heart function, with high sensitivity changes in the right heart function due to its good reproducibility, and it is unaffected by the angle of the sampling line and the flow direction (Herling et al., 2019; Kang et al., 2021; Soveral et al., 2021). The Tei index has been widely used in the evaluation of adult right heart function. The pre-experimental comparative study shows that the measurement error is at a minimum when the scanning speed is adjusted to the maximum, using a TDI mode. The collection of incoming data and preset value in this study adopted this mode. Studies have shown that the variation in the TAPSE value and the Tei index were related to the gestational week (Alvarez & McBrien, 2018; Tague et al., 2017). There was no statistical difference in the gestational week comparison in this study for groups A, B, and C; the measurements of the three groups were comparable. In addition, we performed echocardiography at 90–95 days, to eliminate this measurement error. Excluding the aforementioned factors, this study showed that from late pregnancy to 90 days after birth, the variation in the TAPSE value and the Tei index was correlated with the size of the right heart caused by the oval foramen restriction and premature contraction of the arterial catheter. Hence, the degree of enlargement of the right heart can indirectly reflect the degree of impaired right heart function in the fetal period.

The oval foramen is the entry point of the right atrial blood flow into the left atria during the fetal period; the oval valve and the atrial interval form its outlet. Abnormalities in the inlet and outlet of the oval foramen can limit the blood flow from the right into the left chamber, thus causing the volume overload of the right heart. It is believed that if the right and left atrial channel sare only partially and unintentionally closed; close clinical observation can be considered. The prognosis is generally good after birth; however, cardiac insufficiency is difficult to diagnose during the fetal period (Gu et al., 2018; Lei et al., 2018). The measured TAPSE value in group B decreased in comparison with group A, although the difference between both groups was not significant, indicating that the systolic function of the right heart was decreasing. In contrast, the Tei index in group B increased, and the difference was significant, indicating that the overall function of the right heart was decreasing. Among the 91 patients with oval orifice restriction, six of them did not reach full pregnancy term due to obvious enlargement of the right heart and a small amount of pericardial effusion. The rest were completed at full term, and the fetuses had no clinical abnormalities; however, our study on postnatal babies showed that the differences in the TAPSE value and Tei index for groups A and B were significant. This shows that the changes in the right heart function due to fetal oval for amen restriction still exist at 90 days after birth, although they cause no clinical symptoms. The artery catheter is an important channel for the right blood flow into the pulmonary artery during the fetal period, and it bears about 85% of the right heart shunt volume. The early channel contraction will increase the pressure load and volume load quickly.

Premature contraction of the arterial catheter with fetal edema causes approximately 40% of fetal deaths, before and after birth. Conversely, if the right heart is significantly enlarged without edema, the right ventricular failure shows a poor prognosis (Choi et al., 2013; Lopes et al., 2016; Okada, Muneuchi & Iwaya, 2018). The measurement value of TAPSE decreased between groups A and C, and the difference was significant, indicating that the systolic function of the right heart decreased. In contrast, the Tei index increased in group C, and the difference was significant, indicating that the overall function of the right heart decreased. Among the 68 patients enrolled in this study, gestation ended before the full term in nine cases due to obvious enlargement of the right heart, tricuspid regurgitation increase, and venous catheter A-wave reversal, while the others ended gestation at full term. The proportion of non-term pregnancies in group C was greater than that in group B.

The oxygen saturation was reduced in two newborns after birth, while others showed no abnormal clinical symptoms. We also studied the fetuses after birth of this study; the differences in the TAPSE value and the Tei index for groups A and C were significant, suggesting that changes in the right heart function caused by the premature contraction of the arterial catheter persisted at 90 days after birth, and a few can cause clinical symptoms. However, there was no statistical difference in the proportion of the right heart enlargement for groups B and C in the fetal stage. On this premise, the value was larger in group C compared with the TAPSE and Tei index test values of the other two groups; the difference was significant, showing that the premature contraction of the arterial catheter had a more pronounced impact on the right heart function than the oval foramen restriction. In this study, we used the right heart enlargement after birth as the standard to inspect the accuracy of the diagnostic tests on indicators of the right heart function during the fetal period, and the study results showed that the RV/LV ratio and the Tei index of groups B and C had high diagnostic values in predicting the right heart function after birth. Although the TAPSE value is a stable index that reflects the right heart function, itsuse is limited in the prediction and diagnosis of the right heart function after birth.

### Limitation

In this study, the effects of fetal right heart system enlargement on right heart function after birth were studied and tracked to about 90 days after birth. Since the effect of physiologic pulmonary hypertension on right heart function has disappeared at 90 days of age, measurements of right heart function are reliable. The shortcoming of this study is that it does not elucidate the long-term effects of fetal right heart system enlargement on the right heart function after birth. In the future, cases will be followed up, expected to be followed up to 2 years or more after birth, in order to draw more meaningful conclusions.

### Conclusion

In this study, fetuses were followed up to 90 days after birth, and the study results showed that oval foramen restriction and premature contraction of the arterial catheter would affect the right heart function of fetuses and infants. In addition, we provided the cut-off values for the predictors of the right heart function after birth during the fetal period. Although these findings can provide accurate information for clinical use, the follow-up period was not finished as fetuses have not been followed up for a year or more after birth. Hence, we do not claim to assign any pathological significance to oval foramen restriction and premature contraction of the arterial catheter. Therefore, future studies with a longer follow-up period are warranted to validate our results.

##  Supplemental Information

10.7717/peerj.14702/supp-1 Supplemental Information 1 Patient characteristics

## Additional Information and Declarations

### Data Availability

The following information was supplied regarding data availability: The raw data are available in the Supplemental File.
